# Spatially differentiated expression of quadruplicated green-sensitive RH2 opsin genes in zebrafish is determined by proximal regulatory regions and gene order to the locus control region

**DOI:** 10.1186/s12863-015-0288-7

**Published:** 2015-11-04

**Authors:** Taro Tsujimura, Ryoko Masuda, Ryuichi Ashino, Shoji Kawamura

**Affiliations:** Department of Integrated Biosciences, Graduate School of Frontier Sciences, the University of Tokyo, Kashiwanoha 5-1-5, Kashiwa, 277-8562 Chiba Japan; Department of Advanced Nephrology and Regenerative Medicine, Division of Tissue Engineering, the University of Tokyo Hospital, Hongo 7-3-1, Bunkyo-ku, 113-8655 Tokyo Japan

**Keywords:** Zebrafish, opsin, RH2, Gene duplication, Subfunctionalization, Expression, Gene regulation, RH2-LCR, Gene order

## Abstract

**Background:**

Fish are remarkably diverse in repertoires of visual opsins by gene duplications. Differentiation of their spatiotemporal expression patterns and absorption spectra enables fine-tuning of feature detection in spectrally distinct regions of the visual field during ontogeny. Zebrafish have quadruplicated green-sensitive (RH2) opsin genes in tandem (*RH2-1*, −*2*, −*3*, −*4*), which are expressed in the short member of the double cones (SDC). The shortest wavelength RH2 subtype (*RH2-1*) is expressed in the central to dorsal area of the adult retina. The second shortest wave subtype (*RH2-2*) is expressed overlapping with *RH2-1* but extending outside of it. The second longest wave subtype (*RH2-3*) is expressed surrounding the *RH2–2* area, and the longest wave subtype (*RH2-4*) is expressed outside of the *RH2-3* area broadly occupying the ventral area. Expression of the four RH2 genes in SDC requires a single enhancer (RH2-LCR), but the mechanism of their spatial differentiation remains elusive.

**Results:**

Functional comparison of the RH2-LCR with its counterpart in medaka revealed that the regulatory role of the RH2-LCR in SDC-specific expression is evolutionarily conserved. By combining the RH2-LCR and the proximal upstream region of each RH2 gene with fluorescent protein reporters, we show that the RH2-LCR and the *RH2-3* proximal regulatory region confer no spatial selectivity of expression in the retina. But those of *RH2-1*, −*2* and −*4* are capable of inducing spatial differentiation of expression. Furthermore, by analyzing transgenic fish with a series of arrays consisting of the RH2-LCR and multiple upstream regions of the RH2 genes in different orders, we show that a gene expression pattern related to an upstream region is greatly influenced by another flanking upstream region in a relative position-dependent manner.

**Conclusions:**

The zebrafish RH2 genes except *RH2-3* acquired differential *cis*-elements in the proximal upstream regions to specify the differential expression patterns. The input from these proximal elements collectively dictates the actual gene expression pattern of the locus, context-dependently. Importantly, competition for the RH2-LCR activity among the replicates is critical in this collective regulation, facilitating differentiation of expression among them. This combination of specificity and generality enables seemingly complicated spatial differentiation of duplicated opsin genes characteristic in fish.

**Electronic supplementary material:**

The online version of this article (doi:10.1186/s12863-015-0288-7) contains supplementary material, which is available to authorized users.

## Background

In vertebrates, visual opsins are classified into five phylogenetic types that originated in their common ancestor. RH1 is the rod opsin or rhodopsin responsible for dim-light vision. The other four are cone opsins for color vision: they are SWS1, SWS2, RH2 and M/LWS, and mainly sensitive to UV, blue, green and red light, respectively [1]. These different types of opsin genes are generally expressed in distinct types of photoreceptor cells that are arrayed in the retina to assure color discrimination [2].

Among vertebrates, fish have experienced gain and loss of visual opsins repeatedly by gene duplications and deletions. For example, zebrafish (*Danio rerio*) have ten visual opsin genes: a tandem array of spectrally distinct two LWS genes (*LWS-1* and *LWS-2*) and that of four RH2 genes (*RH2-1*, *RH2-2*, *RH2-3* and *RH2-4*), single-copy SWS1 and SWS2 genes, and two RH1 genes [3, 4]. Medaka (*Oryzias latipes*) [5] and cichlid (Nile tilapia, *Oreochromis niloticus*) [6, 7] have nine and eight opsin genes, respectively.

Newly replicated daughter genes are identical; hence typically only one is likely to be preserved and the others become pseudogenes due to functional redundancy. Nevertheless, if the replicates undergo a process of subfunctionalization, i.e. taking a different part of original function which the ancestral gene had, both genes are more likely to be preserved [8]. The subtype opsin genes in fish indeed achieved the subfunctionalization by differential spatial and temporal expression patterns within the retina as well as divergent absorption spectra of the encoding photopigments [3, 5, 9–14].

In the case of zebrafish, all the four subtypes of RH2 are expressed in the short (or accessory) member of double cones (SDCs) and both two LWS subtypes are expressed in the long (or principal) member of double cones (LDCs) [13, 15, 16]. However, they are differentiated in the expression pattern in the retina [13]. Fish eyes continue to grow through the lifetime by adding new cells to the peripheral zones [17]. Concomitantly, early-expressed subtypes are located centrally in the adult retina. The shortest wavelength RH2 subtype (*RH2-1*) is expressed earliest and in the central to the dorsal area of the adult retina. The second shortest wave subtype (*RH2-2*) is expressed subsequently overlapping with *RH2-1* but extending outside of it. The longer wave RH2 subtype (*RH2-3*) is expressed later and in a region surrounding the *RH2–2* area, and the longest wave RH2 subtype (*RH2-4*) is also expressed later and outside of the *RH2-3* area, broadly occupying the ventral area. Similarly, the shorter wave LWS subtype (*LWS-2*) is expressed earlier and in the central-to-dorsal area in the adult retina, and the longer wave LWS subtype (*LWS-1*) is expressed later in the development and confined peripherally with largely occupying the ventro-nasal area of the adult retina [13]. Thus, in zebrafish, each replicated opsin gene is expressed in a portion of the expression area of their hypothetical ancestral gene, which is presumed to have been expressed throughout the retina, while maintaining the cell-type specificity. As a result, retinal regions that detect spectrally distinct portions of the visual field in the water acquired different spectral sensitivity and presumably different color vision [13].

Medaka and cichlids also show differential expression of tandemly-arrayed opsin genes, which were replicated independently from zebrafish [5, 9–12]. Hence, fish appear to have established regulatory mechanisms for the differential expression of subtype opsins repeatedly. We wondered how fish could accomplish such seemingly complicated regulation for the differential expression in parallel.

We previously showed that, in zebrafish, a single enhancer, named RH2-LCR, was located at the 15-kb upstream of the RH2 gene cluster and was necessary and sufficient for the SDC-specific expression of all the four RH2 genes [18]. In larvae, it was shown that the relative distance from the RH2-LCR to the genes affects their expression levels whereas *RH2-4* is relatively insensitive to the distance effect [18]. We also showed that the two LWS genes of zebrafish are regulated by a single enhancer (LAR) [19]. In the case of LWS genes, the closer gene to LAR, *LWS-1*, is expressed later and more peripherally than *LWS-2* [13]. The proximal upstream region of *LWS-1* appears to have an active role in specifying the spatial expression to the peripheral retina while that of *LWS-2* allows expression throughout the retina in conjunction with LAR. In the presence of the *LWS-1* upstream region, however, the gene expression from the *LWS-2* promoter is excluded from the area where *LWS-1* is expressed [19]. From these observations we hypothesize that the tandemly arrayed genes compete for their interaction with the RH2-LCR/LAR and that the relative distance influences the likeliness, e.g. a closer gene can have a greater chance to interact with it, while a proximal regulatory region can alter this stereotype pattern [19].

In this study, we test the hypothesis for the zebrafish RH2 genes. We first investigate the evolutionary and functional conservation of the RH2-LCR. We further make a series of transgenic fish carrying reporter genes under variously reordered proximal upstream regions from the RH2 genes together with the RH2-LCR.

## Results

### The RH2-LCR regulatory function is evolutionarily conserved

**Fig. 1:**
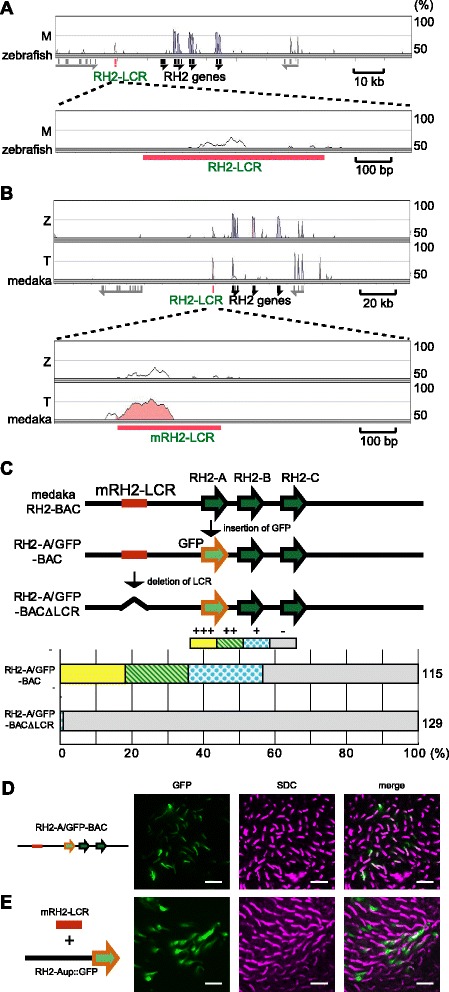
The RH2-LCR is an evolutionarily conserved enhancer in teleosts. **a** Sequence comparison of the RH2 locus between zebrafish and medaka (M) is shown using mVISTA program. The baseline corresponds to the zebrafish RH2 region. An enlarged illustration around the RH2-LCR is also shown. Black and gray bars under the chart are the exons of the RH2 genes and the other genes, respectively. The red bar indicates the RH2-LCR. The sequence homology is indicated to the right of the chart. **b** Sequence comparisons of the RH2 locus of medaka with zebrafish (Z) and *Tetraodon* (T) are shown as in (**a**). Homology regions colored with gray correspond to coding regions of genes and those colored with pink correspond to conserved non-coding sequences. Note that *Tetraodon* has higher homology at the RH2-LCR than zebrafish, reflecting their closer relation with medaka. **c** Construction of the medaka *RH2-A*/GFP-BAC clones (upper panel) and expression levels at 5 dpf of the GFP reporter in zebrafish injected with the BAC clones above (lower panel). The histogram shows the percentage of eyes graded into four levels (+++, ++, + and -) according to the number of GFP-expressing cells in the retina. The names to the left of the histogram indicate the constructs injected. The numbers to the right of the histogram show the total number of eyes examined. **d**, **e** Whole mount retinas of 7-dpf zebrafish embryos injected with the *RH2-A*/GFP-BAC (**d**) and with mixture of the medaka RH2-LCR fragment and the GFP reporter under the 3-kb upstream region of *RH2-A*. GFP fluorescent signals appear as green (*left*) and immunostaining signals of SDCs by the anti-RH2 antibody appear as magenta (*middle*). Overlap of the two signals appears as white. The right panels are the overlays of the left and middle panels. Scale bars = 10 μm

The genomic sequence of the RH2 locus was compared between two superorders Ostariophysi (e.g. zebrafish) and Acanthopterygii (e.g. medaka and *Tetraodon*). Although the RH2 gene duplications are known to have occurred independently in the two superorders [3, 5], a portion of the RH2-LCR was highly conserved among them (Fig. 1a, b). This is consistent with the RH2 opsin genes being expressed in the SDC in both zebrafish and medaka [13, 15, 16, 20].

To test if the sequence conservation reflects its functional importance, we introduced five deletions to the RH2-LCR in the *RH2-1*/GFP-PAC. The *RH2-1*/GFP-PAC is a PAC-vectored clone, modified from the RH2-PAC containing all the four RH2 genes to replace the exon 1 of *RH2-1* with a GFP (green fluorescent protein) reporter gene [18]. The reporter expression in the zebrafish retina was lost only when the central 100 bp of the RH2-LCR was deleted, which corresponds to the region of the highest sequence similarity between species (Additional file 1: Figure S1).

To further test its functional conservation, we introduced the orthologous sequence of medaka to zebrafish for its regulatory activity. We used a BAC-vectored clone from medaka [5] encompassing the orthologous RH2-LCR and all the medaka RH2 genes (*RH2-A*, −*B*, −*C*) in which the exon 1 of *RH2-A* was replaced with GFP. We observed GFP expression in the SDCs in zebrafish (Fig. 1c, d). The removal of the LCR abolished the GFP expression in the retina (Fig. 1c). Consistently, when the medaka RH2-LCR was injected together with the 3-kb upstream region of *RH2-A* conjugated to a GFP reporter, the GFP expression was also observed in the SDCs in zebrafish (Fig. 1e). Thus, the RH2-LCR is an evolutionarily conserved regulatory region that has been present prior to the independent gene duplication events in zebrafish and medaka lineages, and has maintained the regulatory function to drive SDC-specific gene expression.

**Fig. 2:**
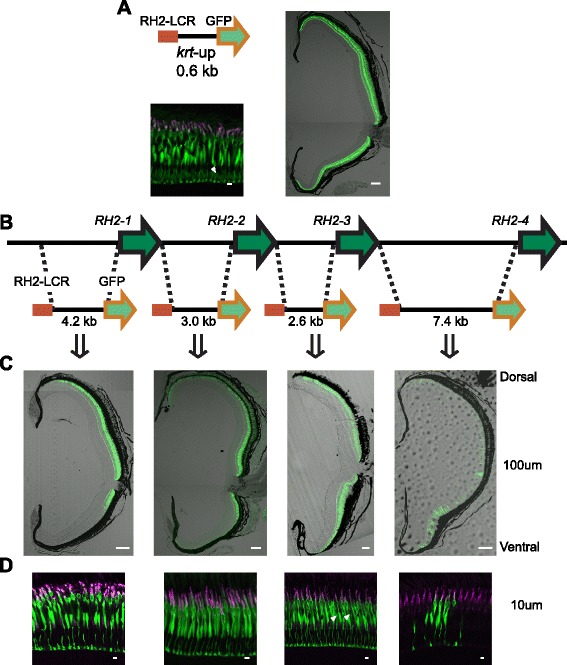
GFP expression patterns specified by the RH2 upstream sequences. **a** The promoter of *keratin 8* attached with the RH2-LCR was linked to a GFP reporter (*top left*). The GFP was mostly expressed in the SDCs with some ectopic expression as indicated by a white arrowhead (*bottom left*). The transverse sections of the retinas of the adult transgenic zebrafish showed GFP expression in the entire region from the dorsal to the ventral retina (*right*). Scale bars = 10 μm (bottom left), 100 μm (*right*). **b** Schematic representation of the RH2 upstream constructs with the RH2-LCR. **c** Images of the transverse sections of the retinas of the adult transgenic zebrafish possessing the respective constructs. The dorsal side is at the top and the ventral side is at the bottom. **d** Vertical sections of the photoreceptor layer in the same adult retinas as in (**c**). Immunostaining signals of SDCs by the anti-RH2 antibody appear as magenta, GFP fluorescent signals appear as green, and overlap of the two signals appears as white. Note the weak ectopic expression by the *keratin 8* and *RH2-3* upstream construct in non-SDC photoreceptor cells as indicated by white arrowheads (**a**, **d**). Scale bars = 100 μm (**c**) and 10 μm (**d**)

When we coupled the RH2-LCR with the proximal upstream region of *keratin 8* as a basal promoter, which presumably has no spatial specificity of expression in the retina [18, 21, 22], the GFP reporter was expressed in all SDCs throughout the retina of the transgenic fish (Fig. 2a). Thus, the RH2-LCR confers no spatial selectivity in the retina on the expression regulation, reflecting its presumed ancestral state prior to gene duplications.

### Roles of proximal regulatory regions in area specificity

We further modified the RH2-PAC to create the *RH2-1*/GFP-*RH2-2*/RFP-PAC and the *RH2-3*/GFP-*RH2-4*/RFP-PAC in which the exon 1 of *RH2-1* or *RH2-3* was replaced with GFP and that of *RH2-2* or *RH2-4* was replaced with RFP (red fluorescent protein), respectively, so that we could visualize the expression pattern of two RH2 genes simultaneously (Additional file 1: Figures S2 and S3). We confirmed that these transgenic zebrafish lines indeed recapitulated the corresponding RH2 genes’ expression as we previously showed using the single-gene replacement constructs (*RH2-1*/GFP-PAC, *RH2-2*/GFP-PAC, *RH2-3*/GFP-PAC, and *RH2-4*/GFP-PAC) [18]. This further confirmed that the RH2-PAC contains the complete set of *cis*-regulatory regions.

We next established transgenic zebrafish lines using only the upstream regions of *RH2-1*, *RH2-2*, *RH2-3* and *RH2-4* coupled respectively with the RH2-LCR and the GFP reporter (Fig. 2b): the upstream sequence of *RH2-1* (4.2 kb) and the entire intergenic regions upstream of *RH2-2* (3.0 kb), of *RH2-3* (2.6 kb), and of *RH2-4* (7.4 kb). The transgenic lines showed that the *RH2-1* and *RH2-2* constructs drove GFP expression confined to the central-to-dorsal area of the retina and the *RH2-4* construct to the ventral area, largely recapitulating the respective expression pattern of these genes (Fig. 2c, see also Additional file 1: Figures S2 and S3) [13]. By contrast, the *RH2-3* construct drove GFP expression in all SDCs of the retina, markedly different from its native narrow expression pattern surrounding the native *RH2-2* expression area (Fig. 2c, see also Additional file 1: Figure S3C, D) [13]. Taken together, the proximal upstream regions of *RH2-1*, −*2* and −*4* are capable of specifying expression area in the retina whereas that of *RH2-3* is not. It was also noted that the transgenic fish with the *RH2-3* construct showed considerable ectopic expression of GFP in non-SDC photoreceptors throughout the retina, while those with the *RH2-1*, −*2* and −*4* constructs did not (Fig. 2d).

### Effect of relative distance to the RH2-LCR among proximal regulatory regions on the spatial expression pattern in the retina

**Fig. 3:**
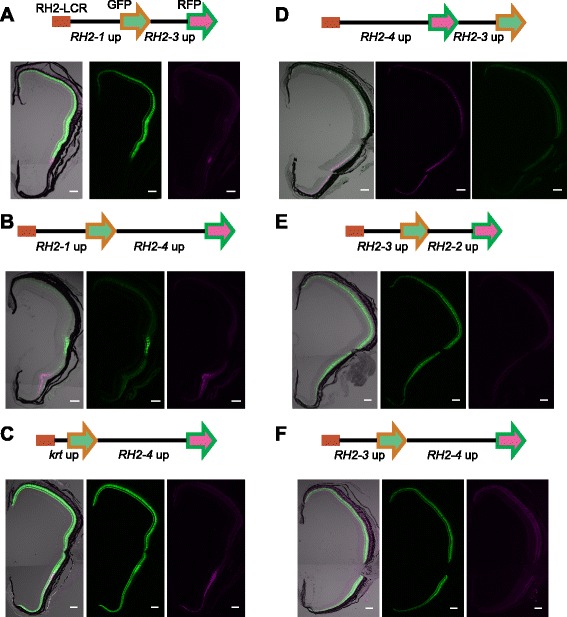
Presence of a competitive promoter modulates reporter expression by the RH2-LCR and RH2 upstream elements. **a**-**f** Schematic representations of constructs with the RH2-LCR and double promoter-reporter sets are depicted at the top. RH2-LCR is represented as a red rectangle. The GFP and RFP reporters are represented as green and magenta arrows, respectively. The upstream sequences used to drive the reporters are indicated below. The lower panels are transverse sections of retinas from the adult transgenic fish with the respective constructs. The middle and right panels are fluorescence from the first and second reporters, respectively. The left is the overlay of the middle and right panels with DIC images of the same retina. The GFP signals appear as green and the RFP signals appear as magenta. Note that the signal in the right panel of (**f**) is autofluorescence from the retinal pigment epithelium as evident in the overlaid image. The dorsal side is at the top, and the ventral side is at the bottom. Scale bars = 100 μm

We then combined two genes’ proximal regulatory regions, each coupled with either GFP or RFP reporter gene, under the RH2-LCR (Fig. 3), and established transgenic zebrafish lines with these constructs. This was to test the previously inferred effect of relative distance to the RH2-LCR among genes [18].

When the *RH2-1* upstream region was placed closer to the RH2-LCR than the other and was combined with either of the *RH2-3*, *RH2-4* or *keratin 8* upstream region (Fig. 3a, b, and Additional file 1: Figure S4A, B), GFP expression driven by the *RH2-1* upstream region was similar to its native pattern, confined in the central-to-dorsal region of the retina. On the other hand, expression pattern of the second gene, represented by RFP expression, varied. The *RH2-3* upstream region drove RFP expression in a narrow area outside the *RH2-1*/GFP area in one transgenic line, similar to its native pattern (Fig. 3a). In another transgenic line with the same construct, the RFP expression was evident in the central-to-dorsal area but weaker in the ventral area (Additional file 1: Figure S4A). Interestingly, the RFP and GFP expression showed reciprocal gradients in intensity, though expression was not totally exclusive (Additional file 1: Figure S4A). Thus, in these two lines, the presence of the *RH2-1* upstream region in front seems to restrict the induction by the *RH2-3* upstream region to the cells in the central-to-dorsal retina that do not or only weakly express GFP. It is of note that the placement of the *RH2-3* upstream region at the second position appears to enhance the expression of the first gene (Fig. 3a). The RFP expression by the *RH2-4* upstream region was similar to its native pattern confined to the ventral area of the retina (Fig. 3b). The RFP expression by the *keratin 8* upstream region was not clearly detected (Additional file 1: Figure S4B).

When the *RH2-4* upstream region was placed after *keratin 8* upstream region, the reporter expression by the *RH2-4* upstream region was again similar to its native pattern confined to the ventral retina while the reporter expression by the *keratin 8* upstream region was in the entire retina (Fig. 3c). When the *RH2-4* upstream region was placed in front of the *RH2-3* upstream region (Fig. 3d), the reporter from the *RH2-4* upstream was expressed strongly in the ventral retina, though also detected elsewhere in the retina. The GFP expression by the *RH2-3* upstream region was weak overall, being almost absent in the ventral area. Thus, the two reporters show reciprocal gradients of expression in this transgenic line (Fig. 3d).

In the above cases, the reporter expression from the *RH2-3* upstream was reshaped by another upstream region located closer to the RH2-LCR (Fig. 3a, d, Additional file 1: Figure S4A). However, when the *RH2-3* upstream region was placed closer to the RH2-LCR than the other (*RH2-2* or *RH2-4* upstream region) (Fig. 3e, f), the reporter expression by the *RH2-3* upstream region was in the entire retina. On the other hand, the reporter gene expression by the *RH2-2* and *RH2-4* upstream regions was not clearly detected (Fig. 3e, f). We speculate, in these cases, the upstream region of *RH2-3* outcompeted those of the other RH2 genes and blocked the access of the RH2-LCR to the downstream.

These results indicate that the RH2 genes’ expression pattern in the retina is not only governed by their own proximal upstream regions but also by those of the other RH2 genes nearby on the array. Importantly, their relative distance (or arrayed order) to the RH2-LCR greatly matters to the effect of the latter.

**Fig. 4:**
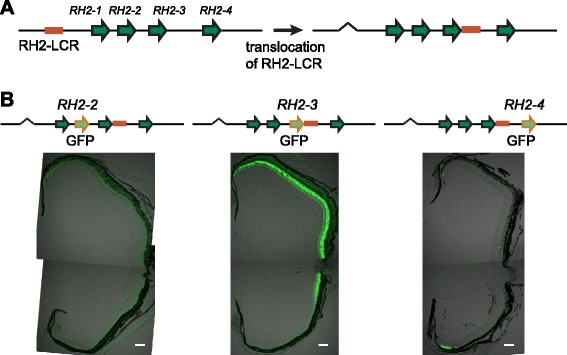
Translocation of the RH2-LCR revealed gene-order dependent competition for the RH2 regulation. **a** Translocation of the RH2-LCR. The RH2-LCR was removed from the original position and then re-inserted into the immediate downstream of *RH2-3* in the RH2-PAC clones [18]. **b** Transverse sections of the adult transgenic zebrafish retinas of the PAC constructs where *RH2-2* (*left*), *RH2-3* (*middle*) and *RH2-4* (*right*) are replaced with the GFP reporter respectively. The GFP signals appear as green. Note that in the left panel the green signal is saturated and only the autofluorescence from the retina is captured. The dorsal side is at the top, and the ventral side is at the bottom. Scale bars = 100 μm

To further test the effect of relative distance with each other in such context-dependent regulation of the RH2 locus, we established transgenic zebrafish lines of modified RH2-PAC clones, in which the RH2-LCR was translocated from the 15-kb upstream of the gene array to the region immediately downstream of *RH2-3* in the RH2-PAC (Fig. 4a). We previously showed that this configuration increased the expression level of *RH2-3* and decreased those of *RH2-1* and *RH2-2* in larvae by transient transgenic assays with GFP reporters [18]. Consistently, in the adult transgenic fish carrying the RH2-PAC clone with GFP replacing *RH2-3*, the expression of *RH2-3*/GFP was clearly extended towards the dorsal area of the retina (Fig. 4b*middle*). By contrast, the expression of *RH2-2*/GFP was abolished (Fig. 4b*left*) and that of *RH2-4*/GFP was maintained in the ventral area and the dorsal tip of the retina in this configuration (Fig. 4b*right*). Thus, upon the translocation, the closer *RH2-3* became the target of the RH2-LCR in the central to dorsal area of the retina. The LCR activity promoting the more distant *RH2-1*/*RH2-2* was reduced. In the ventral retina, *RH2-4* was still predominantly activated, excluding the activation of *RH2-3*.

## Discussion

The present study shows that (i) the regulatory role of the RH2-LCR in SDC-specific expression is evolutionarily conserved; (ii) the RH2-LCR and the *RH2-3* proximal regulatory region provide no spatial selectivity of expression in the retina; (iii) the proximal regulatory regions of *RH2-1*, −*2* and −*4* are capable of inducing spatial differentiation of expression; (iv) these regulatory regions influence with each other to modulate the actual output in a position-dependent manner, which most strikingly determines the spatial pattern of *RH2-3* expression. Since the ancestral RH2 gene was likely expressed in the entire area of the retina, we suggest that the upstream of *RH2-3* maintained the ancestral regulatory feature while those of the others achieved functional modification.

The following lines of evidence support that competition for the interaction with the RH2-LCR among the replicated genes underlies the position-dependent regulation. First, we often observed reciprocal gradients of expression between two reporter genes in our transgenic fish including those in which one is completely repressed (Fig. 3, Additional file 1: Figure S4). Furthermore, genes closer to the RH2-LCR, which should have a higher chance to interact with the enhancer, were more likely and broadly activated, while activation of other genes located further from the LCR was often interrupted (Figs. 3 and 4, Additional file 1: Figure S4).

**Fig. 5:**
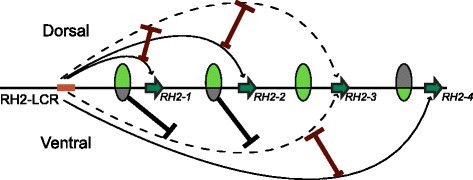
The proposed model of the collective regulation of the RH2 locus in zebrafish. The regulatory activity of the proximal regions in the dorsal and ventral retina is depicted by the top and bottom part of the divided ovals, respectively, at each position. Light green and gray indicate the active and repressive states, respectively. Basically, the RH2-LCR induces gene expression if the upstream region of the target is active as depicted by the arched arrows. However, competition with other members of the locus (whiskered brown bars) and the repressive regulation by the upstream regions of *RH2-1* and *RH2-2* in the ventral retina (black bars) inhibits the interaction of *RH2-3* with the RH2-LCR as indicated by the dashed arrows, restricting its expression into the narrow banded area between the central and ventral retina. Importantly, the dominance in the competitive regulation (indicated by the sizes of the whiskers) greatly depends on the relative position to the RH2-LCR.

However, the competition does not seem to be the only mechanism governing the context-dependent regulation of the RH2 locus. The absence of RFP expression from the *RH2-3* upstream sequence in the ventral retina (Fig. 3a and Additional file 1: Figure S4A) might indicate that the regulatory region upstream of *RH2-1*, which was located between the RH2-LCR and the *RH2-3* upstream in the transgenic lines, represses gene expression in the ventral zone not only of itself but also of *RH2-3* (Fig. 5). It should also be noted that in the double reporter constructs in this study, any promoters including that of *RH2-4* failed to induce gene expression when located downstream of the *RH2-3* promoter due to its blocking activity (Fig. 3e, f). Nevertheless the expression of endogenous *RH2-4*, located downstream of *RH2-3*, is robustly induced in the ventral area (see Additional file 1: Figure S3). This fact implies a mechanism that should interfere the blocking activity by *RH2-3* over *RH2-4*. Perhaps, the repressive action by the upstream of *RH2-1* (and also of *RH2-2*) towards *RH2-3* in the ventral retina might play a role in it (Fig. 5). On the other hand, the translocation of the RH2-LCR to the downstream of *RH2-3* diminished the activation of *RH2-1* [18] and *RH2-2*, while extending the expression of *RH2-3* (Fig. 4). Taken together, these results show that the appropriate positioning of the *cis*-regulatory elements within the locus is crucial for the collective regulation of the quadruplicates.

It should also be noted that the reporter expression induced by the *RH2-3* promoter with the RH2-LCR was not only in the SDCs but also in other types of the photoreceptors. Although such ectopic expression was not clear in the upstream sequences of the other genes, previous studies, in fact, reported that the RH2-LCR sometimes induced weak expression in long single cones, where SWS2 is specifically expressed normally [18, 23]. When reporter genes are integrated in the RH2-PAC clone, however, we have never seen their expression ectopically in non-SDC photoreceptor cells. Therefore, there should be a *cis*-regulatory mechanism that involves not only the RH2-LCR but also other *cis*-elements within the locus to strictly specify the RH2 expression to the SDCs. It can be further speculated that this collective regulation might depend on the genomic context, as was suggested in the *Hoxd* cluster and the *Fgf8* locus, where multiple enhancers cooperatively defines the expression patterns of the target genes [24, 25]. Recently, *sine oculis* homeobox homolog 7 (Six7) was implicated in regulation of the RH2 genes in zebrafish [26]. To deepen our understanding of the cell type-specific regulation, roles of such *trans*-regulatory factors should be studied in parallel with *cis*-regulatory mechanisms.

We asked in this study how the ceaseless duplications and differentiations of the opsin genes in teleosts are accompanied by elaborate building of regulatory mechanism to have the replicates differentially expressed with each other. We found that the competitive regulation between the replicates by a single enhancer plays an important role in the differentiation of the RH2 genes. Remarkably, the LWS in zebrafish utilizes a similar mechanism for their differential expression [19]. We propose that such competitive regulation is advantageous to preserve the replicated opsin genes from pseudogenization in fish, since the competition can intrinsically differentiate and subfunctionalize the replicates by assigning them to a distinct set of the photoreceptor cells. At the same time it precludes a void space in the retina that expresses none of the replicates: without competitive regulation, it might be possible that all lose *cis*-elements necessary to be expressed in some part of the retina through accumulation of mutations in their regulatory regions during the process of differentiation of expression patterns. In fact, it was shown that tandem duplication is the exclusively predominant event in the expansion of the opsin repertoires in fish rather than the whole genome duplication or retroposon-mediated duplications, which do not allow such coordinate regulation in *cis* [27].

In addition, the similarity between the RH2 and LWS in the expression pattern (i.e.. central-to-dorsal vs. ventral) as well as in the *cis*-regulatory mechanisms strongly indicates that the both systems utilize shared *trans*-regulatory components that distinguish different areas of the retina, which might have helped their convergent differentiation. In fact, a recent study revealed that retinoic acid signaling regulates the differential expression of the two LWS genes [28]. Such an extracellular signal might also impact on the regulation of the RH2 genes, though its involvement is still elusive. On the other hand, it should be emphasized that zebrafish also acquired new mutations in the upstream sequences of some subtypes (*RH2-1*, *RH2-2*, *RH2-4* and *LWS-1*) independently for several times to have the spatially polarized differentiation patterns (this study and [19]). This seems to be in a sharp contrast with the duplicated red/green opsin genes in catarrhine primates including human, which have seemingly more or less equivalent promoters with each other, and, as a result, are randomly expressed in the retina in a mutually exclusive way, underlying the trichromacy [29].

The β-globin cluster in human is a well-known example of spatiotemporally-patterned regulation among tandem replicates. The developmental switching of the gene expression along the tandemly clustered genes is also regulated by stage-specific *cis*-elements associated with some of the early-expressed genes together with the competitive regulation that is a function of the gene order [30, 31]. On the other hand, random competition among duplicated genes with apparently equivalent promoters takes place in regulation of olfactory receptor genes to improve the dimension of odor recognition [32–34]. An artificially induced duplication of Protocadherin-α cluster also resulted in stochastic expression of replicates, but not patterned one [35]. These cases might indicate that the only competition among duplicated genes tends to result in stochastic regulation by shared enhancers, and that differential stereotyped spatiotemporal expression further requires additional *cis*-regulatory elements associated with some, but not necessarily all, of the replicates to dictate stage or tissue specificity.

## Conclusions

Our study highlights the differential *cis*-elements embedded in the upstream regions of the RH2 genes as well as their relative distance to the RH2-LCR as fundamental *cis*-regulatory features that collectively shape the differential expression of the quadruplicated opsin genes in zebrafish (Fig. 5). Other fish such as medaka, cichlids, guppy (*Poecilia reticulata*), four-eyed fish (*Anableps anableps*) and barfin flounder (*Verasper moseri*) are also known to have opsin genes duplicated and differentiated in the spatiotemporal expression patterns to adjust their visual sensitivity to heterogeneity in their ontogeny and environment with fine-tuned absorption spectra of the visual pigments [5, 9–12, 27]. Therefore it is anticipated that these differentiated opsins also adopted proximal *cis*-regulatory mutations to differentiate the expression patterns, probably based on concerted regulation through enhancer-sharing generated upon duplications. In order to deepen our understanding of the unique expansion of the opsin repertoires in fish, future studies should further clarify the evolutionary steps of the gene regulatory mechanisms in different lineages.

## Methods

### Sequence comparison of RH2 locus among zebrafish, medaka and *Tetraodon*

The sequence surrounding the zebrafish RH2 locus was obtained from Ensembl genome database of zebrafish, and corresponds to the nucleotide position 32265746–32385745 (120 kb) of chromosome 6 in zebrafish assembly version 6. The sequence surrounding the medaka RH2 locus was obtained from medaka UT genome browser and corresponds to the nucleotide position 1505256–1705255 (200 kb) of scaffold84 of version 1.0. The sequence surrounding the *Tetraodon* RH2 locus was obtained from Ensembl genome database of *Tetraodon*, and corresponds to the nucleotide position 5110001–5140000 (30 kb) of chromosome 11 in *Tetraodon* (*Tetraodon nigroviridis*) assembly version 7 in a reverse orientation. The Sequence alignment between zebrafish, medaka and *Tetraodon* was made with the mVISTA program [36, 37] using the AVID algorithm [38]. Window size was set as 100 bp.

### Usage of zebrafish

All animal protocols were approved by the University of Tokyo animal care and use committee (Approval numbers C-09-02 and C-09-03). The strains of zebrafish (*Danio rerio*) used in the present study were WIK [39] and TL [40], each for microinjection and for mating with transgenic fish of WIK, respectively. They were maintained at 28.5 °C in a 14-h light/10-h dark cycle as described by [41].

### Modification of zebrafish RH2-PAC and medaka RH2-BAC clones

The RH2-PAC and the medaka RH2-BAC (33O2) clones were obtained in [18] and [5], respectively. The insertion of reporter genes and the removal and translocation of the RH2-LCR was all done by the recombineering technique in EL250 [42]. We inserted two I-SceI recognition sites into the vector backbones as described in [19] and [18] in order to facilitate integration of the construct into the genome with the meganuclease [43]. An additional file describes the details of the construction (Additional file 1: Document S1).

### Construction of reporter-expression plasmids

We used pT2GFP-TKPA [19], a derivative from the plasmid clone pT2AL200R150G, which contains the Tol2 transposase recognizing sequences, L200 and R150 [44], as a basal vector backbone for the construction of the GFP expression constructs of the upstream regions of *RH2-1*, −*2*, −*3*, −*4* and *keratin 8*, attached with the RH2-LCR to integrate the transgene via Tol2 transposon-mediated transgenesis. The double reporter constructs with the RH2-LCR were also made in the same vector backbone. An additional file describes the details of the construction (Additional file 1: Document S1).

### Microinjection of DNA constructs into zebrafish embryos

We utilized three different methods of microinjection for transgensis of reporter constructs. We linearized the GFP reporter plasmid of the 3-kb upstream sequence of medaka *RH2-A* by restriction digest with Eco47III to perform co-injection with the linear fragment of medaka RH2-LCR that was amplified by PCR. The co-injection protocol is described in [18, 19]. The plasmid DNAs derived from pT2GFP-TKPA were prepared at final concentration of 25 ng/μl using Plasmid Mini Kit (QIAGEN) or Plasmid Midi Kit (QIAGEN), and were resuspended in 0.1 M KCl and tetramethyl-rhodamin dextran added as a tracer. They were co-injected into the cytoplasm of embryos at the one-cell stage with mRNA of Tol2 transposase of 27 ng/μl that was prepared through *in vitro* transcription from pCS-TP using mMASSAGE mMACHINE kit (Ambion) [44, 45]. The RH2-PAC-derived constructs (20 ng/μl) were injected with I-SceI meganuclease (0.5 units/μl) (New England Biolabs, Beverly, MA) in the solution of 0.5 X commercial meganuclease buffer with tetramethyl-rhodamin dextran tracer [43].

### Establishment of transgenic zebrafish

For the generation of transgenic lines, the injected embryos were grown to sexual maturity and crossed with non-injected fish in a pair-wise fashion. The genomic DNA extracted from a pool of the resulting embryos was examined for the presence of the transgene by PCR amplification of the GFP DNA segment as described in [46]. Importantly, the screening did not rely on presence of the fluorescence in the eyes to avoid biased selection of founder lines. Fish of the subsequent generations were screened again for the presence of the transgene by PCR amplification of the GFP from genomic DNA extracted from the fins. The spatial expression patterns of the reporters in the retina were analyzed in the generation of the offspring from the injected fish (F1) or later.

### Transient assay of GFP expression levels in zebrafish embryos

The injected embryos were grown in 0.003 % 1-phenyl-2-thiourea after 12–24 h post fertilization (hpf) to disrupt pigment formation. Then their eyes were examined at 5 dpf (days post fertilization) for GFP fluorescence under a dissecting fluorescent microscope, and the number of eyes expressing GFP was determined as described by [47]. The eyes were scored as “+++”, “++”, “+”, and “-” when GFP was expressed in more than 50 cells, in 5–50 cells, in 1–4 cells, and in no cells per eye, respectively.

### Immunohistochemistry and image capture

Immunostaining was carried out against the retina sections from adult fish or embryonic whole-mount eye cups, following the protocol described before [47]. Images of GFP, RFP and Cy3 fluorescence were captured using a Zeiss 510 laser-scanning confocal microscope (Carl Zeiss). In case the entire part of the retina sections could not be captured by one image, two overlapping images were collected and then aligned manually to represent the whole retina as a single image.

### Availability of data and materials

All the supporting data are included as additional files.

## Additional file

Additional file 1: Figure S1. Deletion mutagenesis of the RH2-LCR in the *RH2-1*/GFP-PAC. **Figure S2.** Simultaneous recapitulation of the *RH2-1* and *RH2-2* expression by the GFP and RFP reporters. **Figure S3.** Simultaneous recapitulation of the *RH2-3* and *RH2-4* expression by the GFP and RFP reporters. **Figure S4.** The GFP and RFP expression in the transgenic fish of the double promoter-reporter constructs with the RH2-LCR. **Document S1.** Design of reporter constructs used for the transgenesis in the study. (PDF 16104 kb)

## Abbreviations

RH1: Rhodopsin
SWS1: Short-wave-sensitive-1 cone-opsin
SWS2: Short-wave-sensitive-2 cone-opsin
RH2: Rod-opsin-like cone-opsin
LWS: Long-wave-sensitive cone-opsin
SDC: Short member of double cones
LDC: Long member of double cones
RH2-LCR: RH2-locus control region
LAR: LWS activating region
PAC: P1-derived artificial chromosome
BAC: Bacterial artificial chromosome
GFP: Green fluorescent protein
RFP: Red fluorescent protein
Hpf: Hours post fertilization
Dpf: Days post fertilization

## References

[1] Yokoyama S (2000). Molecular evolution of vertebrate visual pigments. Prog Retin Eye Res.

[2] Kawamura S, Inoue-Murayama M, Kawamura S, Weiss A (2011). Evolutionary diversification of visual opsin genes in fish and primates. From genes to animal behavior: Social structures, personalities, communication by color.

[3] Chinen A, Hamaoka T, Yamada Y, Kawamura S (2003). Gene duplication and spectral diversification of cone visual pigments of zebrafish. Genetics.

[4] Morrow JM, Lazic S, Chang BS (2011). A novel rhodopsin-like gene expressed in zebrafish retina. Vis Neurosci.

[5] Matsumoto Y, Fukamachi S, Mitani H, Kawamura S (2006). Functional characterization of visual opsin repertoire in Medaka (*Oryzias latipes*). Gene.

[6] Carleton KL, Kocher TD (2001). Cone opsin genes of African cichlid fishes: Tuning spectral sensitivity by differential gene expression. Mol Biol Evol.

[7] Spady TC, Parry JW, Robinson PR, Hunt DM, Bowmaker JK, Carleton KL (2006). Evolution of the cichlid visual palette through ontogenetic subfunctionalization of the opsin gene arrays. Mol Biol Evol.

[8] Zhang J (2003). Evolution by gene duplication: an update. Trends Ecol Evol.

[9] Carleton KL, Spady TC, Streelman JT, Kidd MR, McFarland WN, Loew ER (2008). Visual sensitivities tuned by heterochronic shifts in opsin gene expression. BMC Biol.

[10] Hofmann CM, Carleton KL (2009). Gene duplication and differential gene expression play an important role in the diversification of visual pigments in fish. Integr Comp Biol.

[11] Owens GL, Rennison DJ, Allison WT, Taylor JS (2012). In the four-eyed fish (*Anableps anableps*), the regions of the retina exposed to aquatic and aerial light do not express the same set of opsin genes. Biol Lett.

[12] Rennison DJ, Owens GL, Allison WT, Taylor JS (2011). Intra-retinal variation of opsin gene expression in the guppy (*Poecilia reticulata*). J Exp Biol.

[13] Takechi M, Kawamura S (2005). Temporal and spatial changes in the expression pattern of multiple red and green subtype opsin genes during zebrafish development. J Exp Biol.

[14] Davies WI, Collin SP, Hunt DM (2012). Molecular ecology and adaptation of visual photopigments in craniates. Mol Ecol.

[15] Raymond PA, Barthel LK, Rounsifer ME, Sullivan SA, Knight JK (1993). Expression of rod and cone visual pigments in goldfish and zebrafish: a rhodopsin-like gene is expressed in cones. Neuron.

[16] Vihtelic TS, Doro CJ, Hyde DR (1999). Cloning and characterization of six zebrafish photoreceptor opsin cDNAs and immunolocalization of their corresponding proteins. Vis Neurosci.

[17] Stenkamp DL (2007). Neurogenesis in the fish retina. Int Rev Cytol.

[18] Tsujimura T, Chinen A, Kawamura S (2007). Identification of a locus control region for quadruplicated green-sensitive opsin genes in zebrafish. Proc Natl Acad Sci U S A.

[19] Tsujimura T, Hosoya T, Kawamura S (2010). A single enhancer regulating the differential expression of duplicated red-sensitive opsin genes in zebrafish. PLoS Genet.

[20] Hisatomi O, Satoh T, Tokunaga F (1997). The primary structure and distribution of killifish visual pigments. Vision Res.

[21] Gong Z, Ju B, Wang X, He J, Wan H, Sudha PM (2002). Green fluorescent protein expression in germ-line transmitted transgenic zebrafish under a stratified epithelial promoter from *keratin8*. Dev Dyn.

[22] Parinov S, Kondrichin I, Korzh V, Emelyanov A (2004). *Tol2* transposon-mediated enhancer trap to identify developmentally regulated zebrafish genes in vivo. Dev Dyn.

[23] Fang W, Bonaffini S, Zou J, Wang X, Zhang C, Tsujimura T (2013). Characterization of transgenic zebrafish lines that express GFP in the retina, pineal gland, olfactory bulb, hatching gland, and optic tectum. Gene Expr Patterns.

[24] Montavon T, Soshnikova N, Mascrez B, Joye E, Thevenet L, Splinter E (2011). A regulatory archipelago controls Hox genes transcription in digits. Cell.

[25] Marinic M, Aktas T, Ruf S, Spitz F (2013). An integrated holo-enhancer unit defines tissue and gene specificity of the Fgf8 regulatory landscape. Dev Cell.

[26] Ogawa Y, Shiraki T, Kojima D, Fukada Y (2015). Homeobox transcription factor Six7 governs expression of green opsin genes in zebrafish. Proc Biol Sci.

[27] Rennison DJ, Owens GL, Taylor JS (2012). Opsin gene duplication and divergence in ray-finned fish. Mol Phylogenet Evol.

[28] Mitchell DM, Stevens CB, Frey RA, Hunter SS, Ashino R, Kawamura S (2015). Retinoic Acid signaling regulates differential expression of the tandemly-duplicated long wavelength-sensitive cone opsin genes in zebrafish. PLoS Genet.

[29] Smallwood PM, Wang Y, Nathans J (2002). Role of a locus control region in the mutually exclusive expression of human red and green cone pigment genes. Proc Natl Acad Sci U S A.

[30] Hanscombe O, Whyatt D, Fraser P, Yannoutsos N, Greaves D, Dillon N (1991). Importance of globin gene order for correct developmental expression. Genes Dev.

[31] Tanimoto K, Liu Q, Bungert J, Engel JD (1999). Effects of altered gene order or orientation of the locus control region on human beta-globin gene expression in mice. Nature.

[32] Fuss SH, Omura M, Mombaerts P (2007). Local and cis effects of the H element on expression of odorant receptor genes in mouse. Cell.

[33] Nishizumi H, Kumasaka K, Inoue N, Nakashima A, Sakano H (2007). Deletion of the core-H region in mice abolishes the expression of three proximal odorant receptor genes in cis. Proc Natl Acad Sci U S A.

[34] Serizawa S, Miyamichi K, Nakatani H, Suzuki M, Saito M, Yoshihara Y (2003). Negative feedback regulation ensures the one receptor-one olfactory neuron rule in mouse. Science.

[35] Kaneko R, Abe M, Hirabayashi T, Uchimura A, Sakimura K, Yanagawa Y (2014). Expansion of stochastic expression repertoire by tandem duplication in mouse Protocadherin-alpha cluster. Sci Rep.

[36] Frazer KA, Pachter L, Poliakov A, Rubin EM, Dubchak I (2004). VISTA: Computational tools for comparative genomics. Nucleic Acids Res.

[37] Mayor C, Brudno M, Schwartz JR, Poliakov A, Rubin EM, Frazer KA (2000). VISTA: Visualizing global DNA sequence alignments of arbitrary length. Bioinformatics.

[38] Bray N, Dubchak I, Pachter L (2003). AVID: A global alignment program. Genome Res.

[39] Rauch G-J, Granato, M., and Haffter, P.: A polymorphic zebrafish line for genetic mapping using SSLPs on high-percentage agarose gels. Tech Tips Online 1997, T01208.

[40] Haffter P, Odenthal J, Mullins MC, Lin S, Farrell MJ, Vogelsang E (1996). Mutations affecting pigmentation and shape of the adult zebrafish. Dev Genes Evol.

[41] Westerfield M (1995). The zebrafish book: A guide for the laboratory use of zebrafish (*Danio Rerio*).

[42] Lee EC, Yu D, Martinez de Velasco J, Tessarollo L, Swing DA, Court DL (2001). A highly efficient *Escherichia coli*-based chromosome engineering system adapted for recombinogenic targeting and subcloning of BAC DNA. Genomics.

[43] Thermes V, Grabher C, Ristoratore F, Bourrat F, Choulika A, Wittbrodt J (2002). *I-SceI* meganuclease mediates highly efficient transgenesis in fish. Mech Dev.

[44] Urasaki A, Morvan G, Kawakami K (2006). Functional dissection of the *Tol2* transposable element identified the minimal *cis*-sequence and a highly repetitive sequence in the subterminal region essential for transposition. Genetics.

[45] Kawakami K, Takeda H, Kawakami N, Kobayashi M, Matsuda N, Mishina M (2004). A transposon-mediated gene trap approach identifies developmentally regulated genes in zebrafish. Dev Cell.

[46] Hamaoka T, Takechi M, Chinen A, Nishiwaki Y, Kawamura S (2002). Visualization of rod photoreceptor development using GFP-transgenic zebrafish. Genesis.

[47] Luo W, Williams J, Smallwood PM, Touchman JW, Roman LM, Nathans J (2004). Proximal and distal sequences control UV cone pigment gene expression in transgenic zebrafish. J Biol Chem.

