# Supplementary material for: Spatially differentiated expression of quadruplicated green-sensitive RH2 opsin genes in zebrafish is determined by proximal regulatory regions and gene order to the locus control region
Source: BMC Genet. 2015 Nov 4;16:130. doi: 10.1186/s12863-015-0288-7 (PMC4634787; doi:10.1186/s12863-015-0288-7)
Supplement: Additional file 1: Figure S1. — Deletion mutagenesis of the RH2-LCR in the RH2-1/GFP-PAC. Figure S2. Simultaneous recapitulation of the RH2-1 and RH2-2 expression by the GFP and RFP reporters. Figure S3. Simultaneous recapitulation of the RH2-3 and RH2-4 expression by the GFP and RFP reporters. Figure S4. The GFP and RFP expression in the transgenic fish of the double promoter-reporter constructs with the RH2-LCR. Document S1. Design of reporter constructs used for the transgenesis in the study. (PDF 16104 kb) [file 12863_2015_288_MOESM1_ESM.pdf]

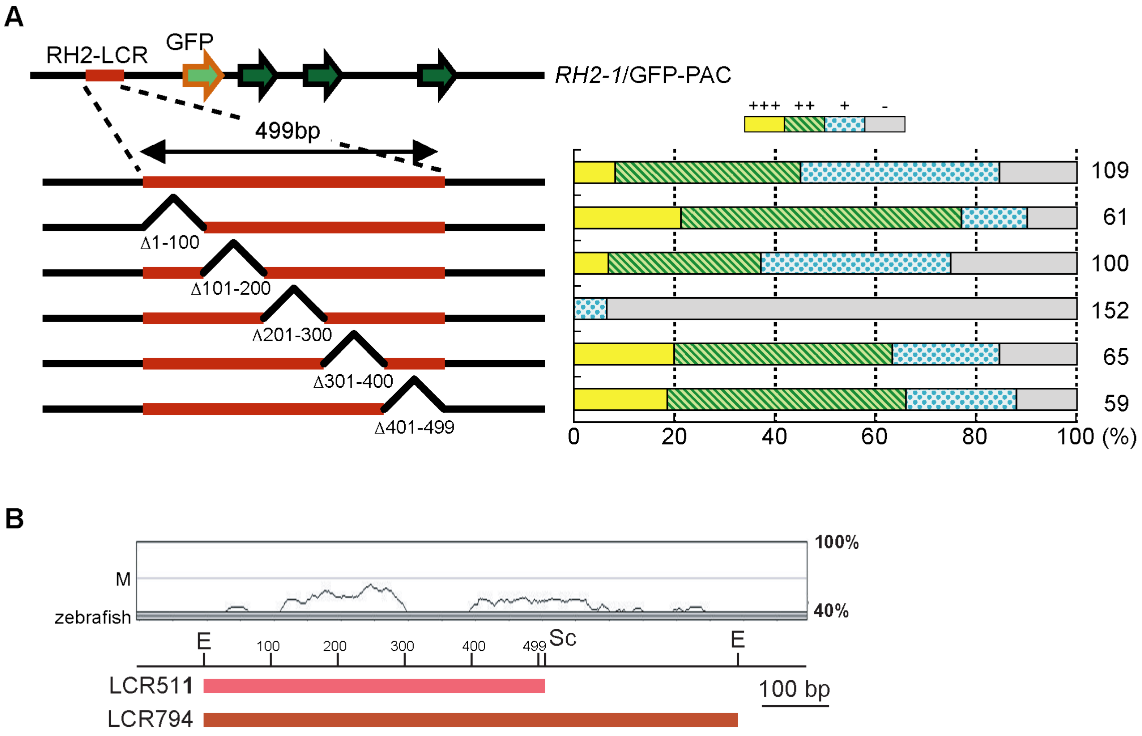

Figure S1

Deletion mutagenesis of the RH2-LCR in the *RH2-1*/GFP-PAC. (A) (Left) The deletion mutants of the RH2-LCR are schematically depicted. Every 100 bp of the RH2-LCR was serially deleted from the *RH2-1*/GFP-PAC construct. (Right) The histograms showed the expression levels of the GFP reporter in 5-dpf zebrafish injected with the constructs indicated to the left. The histogram shows the percentage of eyes graded into four levels (+++, ++, + and -) according to the number of GFP-expressing cells in the retina. Only the deletion of the central 100 bp led to significant reduction in GFP expression. (B) The mVISTA plot along the RH2-LCR region between zebrafish and medaka. The light and dark red bars at the bottom represents the 511-bp region used in [1] (EcoRI/SacI) and the 794-bp region used in the present study (EcoRI/EcoRI) for transgenesis of the reporter constructs, respectively. The coordinates corresponding to the deletion breakpoints in (A) are also plotted along the x-axis.

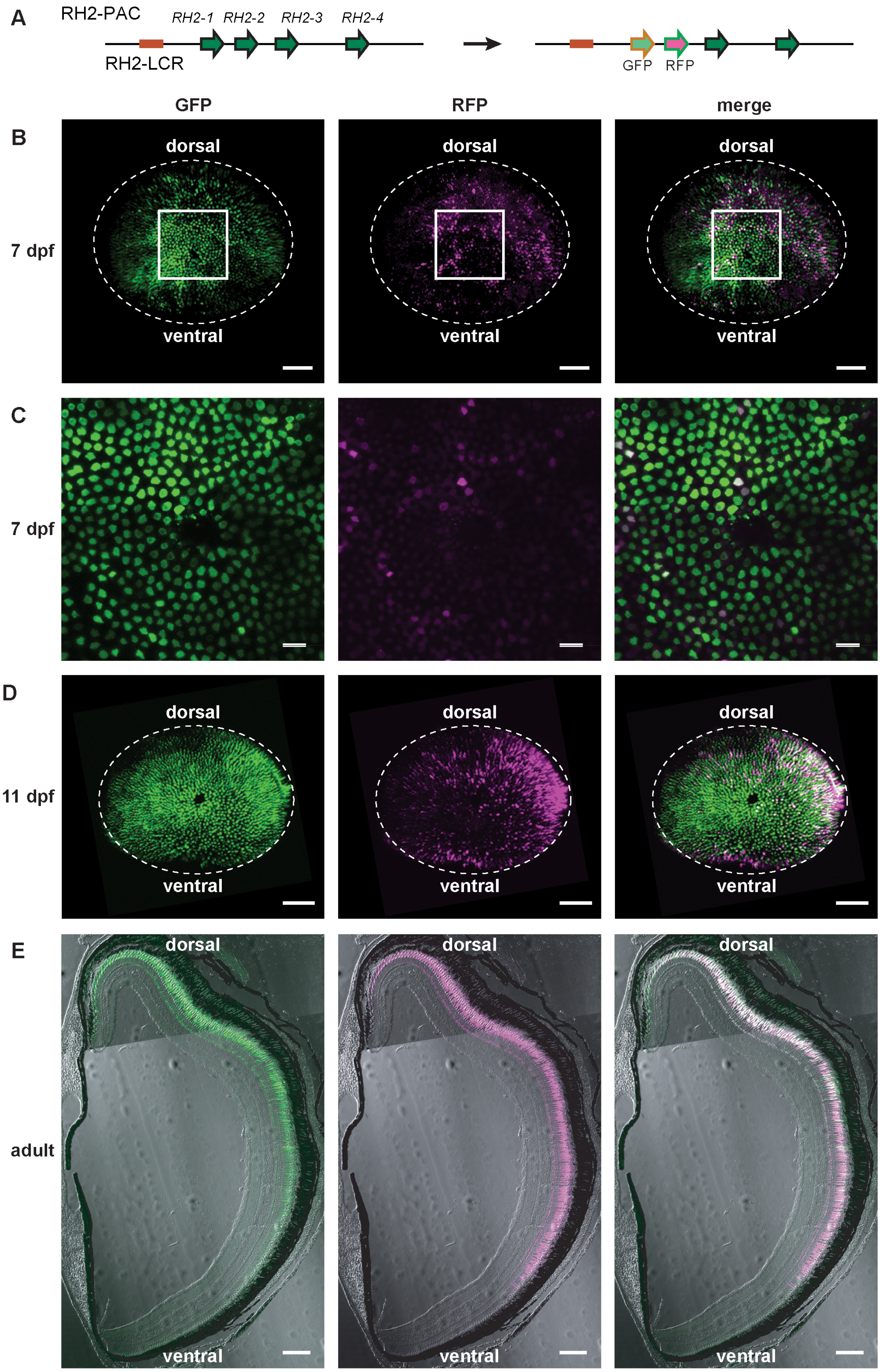
A
RH2-PAC
RH2-1 RH2-2 RH2-3 RH2-4
RH2-LCR
GFP RFP
GFP
RFP
merge
B
7 dpf
dorsal
ventral
C
7 dpf
D
11 dpf
dorsal
ventral
E
adult
dorsal
ventral

Figure S2

Simultaneous recapitulation of the *RH2-1* and *RH2-2* expression by the GFP and RFP reporters. (A) GFP and RFP reporters were inserted in the place of *RH2-1* and *RH2-2*, respectively, in the RH2-PAC clone. (B-E) The reporter expression was analyzed in the transgenic fish carrying the RH2-PAC construct of (A). The GFP signals appear as green and the RFP signals appears as magenta. The overlay of the two signals appears as white. The right panels are the overlays of the left and the middle panels. (B) A whole mount retina of a 7-dpf fish. Images are superimposed view of stacked sectional images serially obtained using confocal laser scanning microscopy. The dorsal side is at the top. (C) Expanded views of the central area of a whole mount retina shown in (B). (D) A whole mount retina of an 11-dpf fish as shown in (B). (E) A transverse section of the adult retina. The dorsal side is at the top and the ventral side is at the bottom. Scale bars = 50 μm (B, D), 10 μm (C) and 100 μm (E).

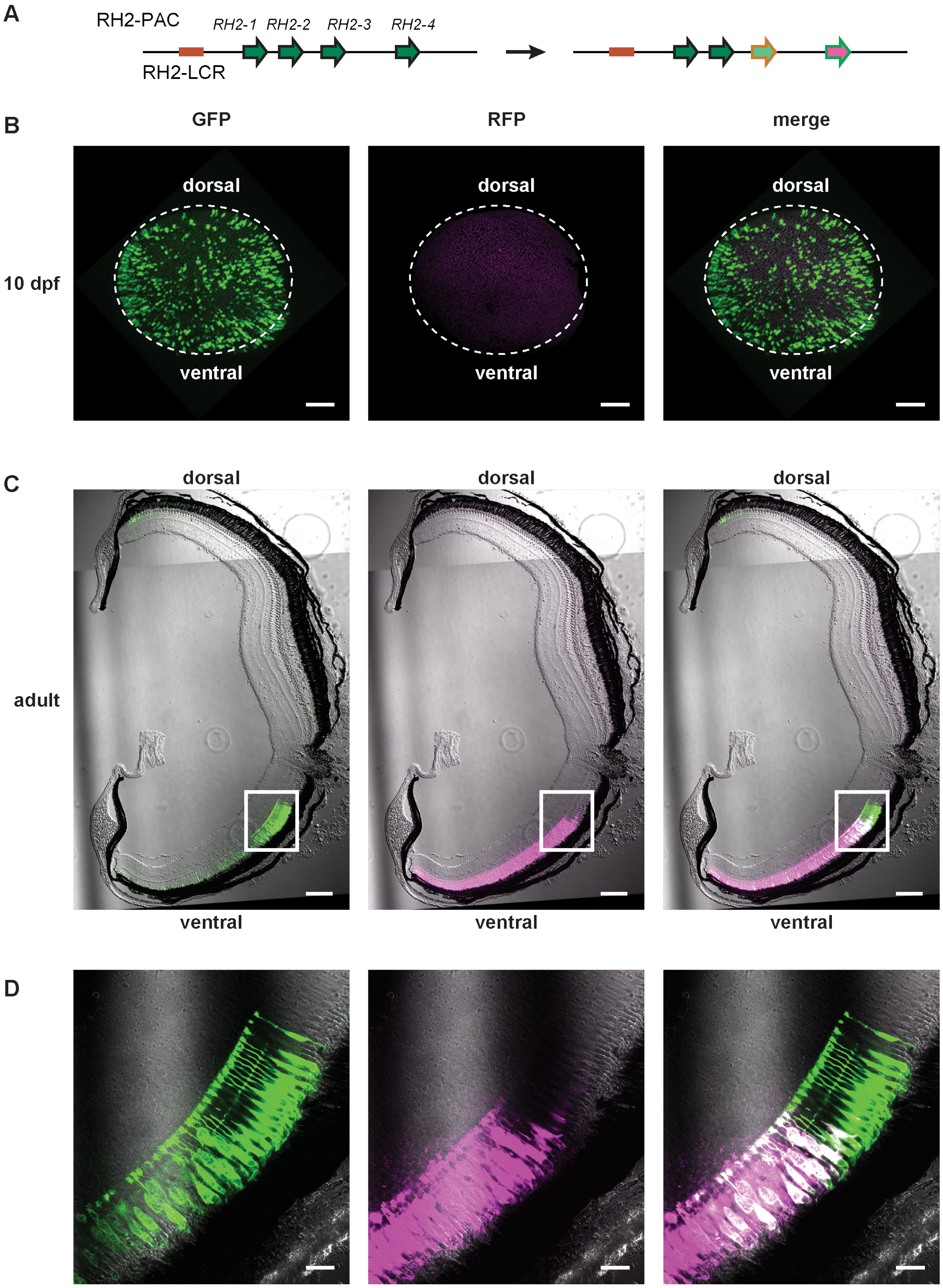
A
RH2-PAC
RH2-1
RH2-2
RH2-3
RH2-4
RH2-LCR
B
GFP
RFP
merge
10 dpf
dorsal
ventral
C
adult
dorsal
ventral
D

Figure S3

Simultaneous recapitulation of the *RH2-3* and *RH2-4* expression by the GFP and RFP reporters. (A) GFP and RFP reporters were inserted in the place of *RH2-3* and *RH2-4*, respectively, in the RH2-PAC clone. (B-D) The images of the retina of the transgenic fish carrying the RH2-PAC construct of (A). The GFP signals appear as green and the RFP signals appear as magenta. The overlay of the two signals appears as white. The right panels are the overlays of the left and the middle panels. (B) A whole mount retina of a 10-dpf fish. Images are superimposed view of stacked sectional images serially obtained using confocal laser scanning microscopy. The dorsal side is at the top. (C) A transverse section of the adult retina. The dorsal side is at the top and the ventral side is at the bottom. (D) Expanded views of (C). There are photoreceptor cells expressing both GFP and RFP. Scale bars = 50 μm (B), 100 μm (C) and 20 μm (D).

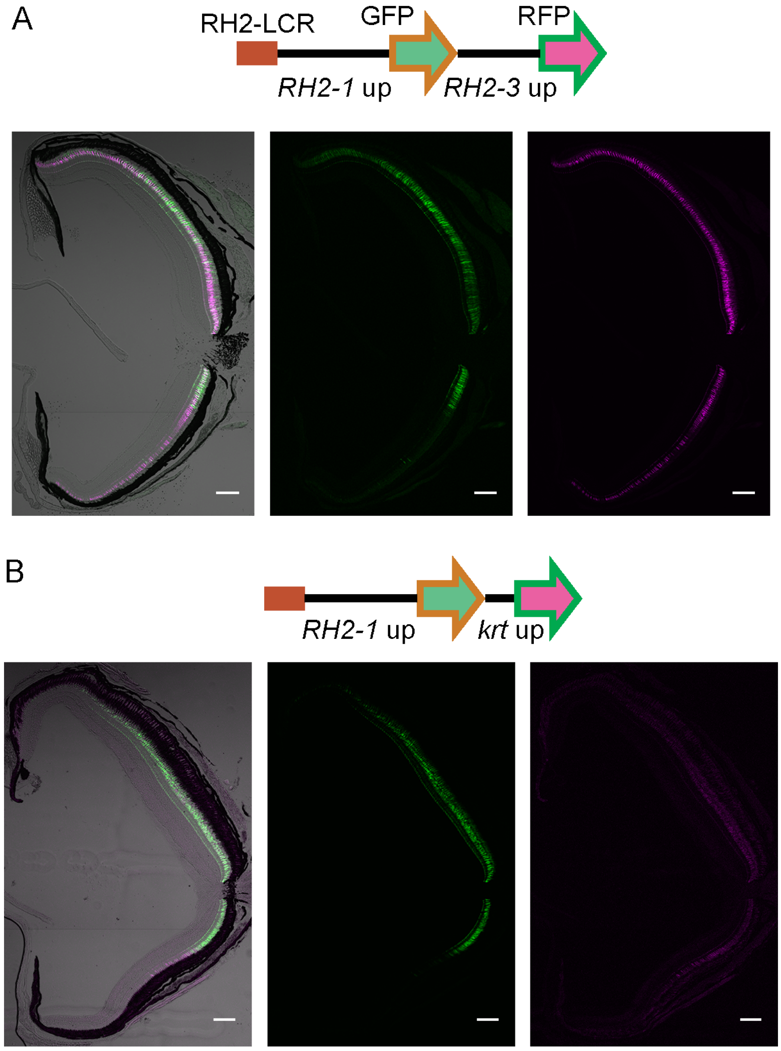
A
RH2-LCR
GFP
RFP
RH2-1 up
RH2-3 up
B
RH2-1 up
krt up

Figure S4

The GFP and RFP expression in the transgenic fish of the double promoter-reporter constructs with the RH2-LCR. (A-B) Schematic representations of constructs with the RH2-LCR and double promoter-reporter pairs are depicted at the top. The RH2-LCR is represented as a red rectangle. The GFP and RFP reporters are depicted as green and magenta arrows, respectively. The upstream sequences used to drive the reporters are indicated below. The lower panels are transverse sections of retinas from the adult transgenic fish carrying the respective constructs. The middle panel is fluorescence from the first reporter and the right one is that from the second reporter. The left is the overlay of the middle and right panels with DIC images of the same retina. The GFP signals appear as green and the RFP signals appear as magenta. The dorsal side is at the top, and the ventral side is at the bottom. Scale bars = 100 μm.

Document S1

Design of reporter constructs used for the transgenesis in the study.

Recombineering of zebrafish RH2-PAC and medaka RH2-BAC clones.

The RH2-PAC and the medaka RH2-BAC (33O2) clones were obtained in [1] and [2], respectively. We inserted two I-SceI recognition sites into the vector backbones as described in [1] and [3] in order to facilitate integration of the construct into the genome with the meganuclease. Briefly, homologous sequences of c.a. 50-bp for the recombination were attached to a cassette consisting of two I-SceI sites flanking an ampicillin-resistant gene with PCR using primers listed below from the template plasmid harboring the I-SceI-Amp^r-I-SceI cassette [3].

PCR primers for amplification of the targeting cassette of I-SceI-Amp^r-I-SceI.

Name	Sequence (5’ > 3’)
zebrafish-PAC::I-SceI-Amp^r-FWD	GCGCTGAGGTCTGCCTCGTGAAGAAGGTGTTGCTG ACTCATACCAGCAGGAATTCGATTAGGGATAAC
zebrafish-PAC::I-SceI-Amp^r-REV	AAAGCCACGTTGTGTCTCAAAATCTCTGATGTTAC ATTGCACAAGTCGATAAGCTTGATATTACCCTG
medaka-BAC::I-SceI-Amp^r-FWD	GGCCGCCCGGGCCGTCGACCAATTCTCATGTTTGA CAGCTTATCAGCAGGAATTCGATTAGGGATAAC
medaka-BAC::I-SceI-Amp^r-REV	CTGTCCTTCCTGGCGACGGTTACGCCGCTCCATGAG CTTATCGCTCGATAAGCTTGATATTACCCTG

Then the targeting cassettes were subject to the recombination in EL250 containing the PAC or BAC clones, replacing the kanamycin- or chloramphenicol-

resistant gene of the vector backbones of the zebrafish RH2-PAC or the medaka RH2-BAC clones, respectively. The GFP and RFP reporter genes were also introduced with the recombination system. We PCR-amplified the GFP-CAT cassette and RFP-Km^r cassette [3] with the primers listed below and employed the fragments for the recombination, followed by the screening of positive clones by the drug selection.

PCR primers for amplification of the GFP-CAT and RFP-Km^{r} cassettes targeting the first exon of zebrafish *RH2-1*, *RH2-2*, *RH2-3* and *RH2-4*, and medaka *RH2-A*.

Name	Sequence (5’ > 3’)
RH2-1<>GFP-CAT-FWD	ATTCATATTTCCACATAGGCGGTTTTAATTTAGGTTTCAGTAAAAATGGTGAGCAAGGGCGAGG
RH2-1<>GFP-CAT-REV	CATGTACTGCATTCAAATCTATGCACTGCTAGTTACTCCTTACCTAACTGGCCTCAGGCATTTG
RH2-2<>GFP-CAT-FWD	GTTTTGCTGGTGTCTTCTTGGCATACGTGCAACACCTCCAGCAGGATGGTGAGCAAGGGCGAGGAG
RH2-2<>GFP-CAT-REV	TGAAACAATAAAATAAACGAATTCCTGCTGAAATATTCTTACCTAACTGGCCTCAGGCATTTGAG
RH2-3<>GFP-CAT-FWD	AACAGATTGCATCCTCCTCGTGTTCTGGATCACTAGCAGGCAGAGATGGTGAGCAAGGGCGAGGAG
RH2-3<>GFP-CAT-REV	TCAAATAAATCCAAAAGGTAAACTGCAGAACAATTATCCTCACCTAACTGGCCTCAGGCATTTGAG
RH2-4<>GFP-CAT-FWD	AGCAGGTTACATCTTTTCATCCATCTGGATCTTTAGCAGGTAGAGATGGTGAGCAAGGGCGAGGAG
RH2-4<>GFP-CAT-REV	ATCTACTAGCGGTGTATATTGTCAACTAGGCTAAATAAGA(G/T)CTCACCTAACTGGCCTCAGGCATTTGAG
RH2-2<>RFP-Km^{r}-FWD	GTTTTGCTGGTGTCTTCTTGGCATACGTGCAACACCTCCAGCAGGATGGCCTCCTCCGAGGACGT
RH2-2<>RFP-Km^{r}-REV	TGAAACAATAAAATAAACGAATTCCTGCTGAAATATTCTTACCTCCTGAATCGCCCCATCATCC
RH2-4<>RFP-Km^{r}-FWD	AGCAGGTTACATCTTTTCATCCATCTGGATCTTTAGCAGGTAGAGATGGCCTCCTCCGAGGACGT
RH2-4<>RFP-Km^{r}-REV	ATCTACTAGCGGTGTATATTGTCAACTAGGCTAAATAAGA(G/T)CTCACCTCCTGAATCGCCCCATCATCC
RH2-A<>GFP-CAT-FWD	TCAACAGTAACTCTGCTTGGCTAATCCAGATCCTAACTTGCAAAGATGGTGAGC AAGGGCGAGG
RH2-A<>GFP-CAT-REV	CCATCAGCCACGATTTGCTCTTAAGCATTTTGCATCAGTTTACCTAACTGGCCTCAGGCATTTG

Deletion and translocation of the RH2-LCR in the RH2-PAC clone.

The RH2-LCR was PCR-amplified from the RH2-PAC clone with its 5’ and 3’ flanking sequences of ~200 bp each, designated 5FL and 3FL, respectively, by the primers listed below, and was cloned into the EcoRV site of pBluescript II (SK-) plasmid. The EcoRI site had been removed from the plasmid before the insertion. The total length of 5FL-LCR-3FL was 909 bp. A Km^r gene flanked by the FRT sequences at both sides, FRT-Km^r-FRT, was inserted into the EcoRI site of the RH2-LCR in the plasmid. The resulting cassette was designated as 5FL-FRT-Km^r-FRT-LCR-3FL. FRT-Km^r-FRT was also PCR-amplified from pCYPAC6 by the primers below.

Primers for PCR amplification of 5FL-LCR-3FL and FRT-Km^r-FRT. The primers for the latter were attached to the recognition site of EcoRI at the 5’ ends.

Name	Sequence (5’ > 3’)
5FL-LCR-FWD	TCTGGAGTTTAACAAAGAGACTC
3FL-LCR-REV	CCTTGCAAGCTTTATGATACAGC
FRT-Km^r-FWD	AGGAATTCGAAGTTCCTATTCTCTAGAAAGTATAGGAACT TCTCTCAAAATCTCTGATGTTAC
FRT-Km^r-REV	AGGAATTCGAAGTTCCTATACTTTCTAGAGAATAGGAACT TCCCTGAATCGCCCCATCATC

To prepare deletion mutants of the RH2-LCR in the RH2-PAC clone, the RH2-LCR was removed from the 5FL-FRT-Km^r-FRT-LCR-3FL plasmid by PCR amplification directing outside from the RH2-LCR and subsequent self-reconnection of the resulted FRT and 3FL ends. The assembled cassette, 5FL-FRT-Km^r-FRT-3FL, was isolated from the plasmid by PCR and subjected to site-specific recombination to replace the RH2-LCR in the RH2-PAC clone with the FRT-Km^r-FRT cassette. In

EL250, induction of *flpe* resulted in the excision of the FRT-Km^r-FRT cassette leaving one FRT sequence [4].

When the RH2-LCR was partially deleted from *RH2-1*/GFP-PAC, the same strategy was employed as above for the complete deletion. The primer sets designed for the generation of deletion mutants of the RH2-LCR are listed below.

Primers used for full and partial deletions of the RH2-LCR. The nucleotide positions within the RH2-LCR in the 5FL-FRT-Km^r-FRT-LCR-3FL plasmid was numbered from +1 to +499 (see Figure S1). The numbers in the names of the following primers indicate the position of the 5' and the 3' ends of the primers.

Name	Sequence (5' > 3')
LCR:-1--23-REV	GAATTCGAAGTTCCTATACTTTC
LCR:101-118-FWD	CTTCGACATGCTGAAGGC
LCR:100-81-REV	GACCAGAGCCAAATGAAGAC
LCR:201-221-FWD	CAGCTTGTCAGGGCCTTTTTG
LCR:200-164-REV	TTAACACTATTTCTTGTTTGCATCCTCTCCTAATACC
LCR:301-319-FWD	TCTGACAGACGGGACAGGC
LCR:300-280-REV	TTAACATCTCTGAAGTGTGTC
LCR:401-426-FWD	ATAACATATACTCTGCATATAATAAG
LCR:400-378-REV	ATGACTTTTTTAAAGGTCATGG
LCR:500-519-FWD	GAGCTCTAAAGTGCATGATT

For convenience sake, to translocate the RH2-LCR in the RH2-PAC clones, the 5FL-FRT-Km^r-FRT-LCR-3FL plasmid was utilized as a source of Km^r linked to the RH2-LCR for a drug selection purpose. The Km^r-FRT-LCR cassette was PCR-amplified from the plasmid by the primers below, though the FRT in the cassette was irrelevant for this construction. To insert the cassette to the 3' untranslated region (UTR) of *RH2-3* by the homologous recombination, the forward PCR primer contained the 3' UTR sequence after the stop codon of *RH2-3*, and the reverse primer contained their adjacent downstream sequences, respectively. The homologous recombination with the amplified cassette was conducted against the RH2-PAC clones that had GFP insertion into the positions of either *RH2-2*, *RH2-3* and *RH2-4*, adding the Km^r-FRT sequence remaining at the recombination site together.

Primers for PCR-amplification of the targeting cassettes of Km^r-FRT-LCR for its insertion into the downstream of *RH2-3*.

Name	Sequence (5' > 3')
LCR>(3)-FWD	AAAATGGATAGGAATAAATACTGGCATTAAAAGTGTTTG TGTTTTTCTCAAAATCTCTGATGTTACATTG
LCR>(3)-REV	TGTGTCTGTGAACAAACTGAAGATTAAATCAGTAAATGA ATAATGTTACAAAATGTAAAAAGTGTCATAC

Deletion of medaka RH2-LCR from the RH2-BAC.

The LCR sequence was simply replaced by the kanamycin-resistant gene through homologous recombination in EL250. The primer pair to obtain the targeting cassette is shown below.

Primers for PCR amplification of FRT-Km^r-FRT to delete the medaka RH2-LCR from the BAC clone.

Name	Sequence (5’ > 3’)
medaka LCR<>Km^r-FWD	GACAGGAGTCACCTCTAACATTTCCAGACTTCTTAGCCTA<br>CTTATTCTCAAAATCTCTGATGTTACATTG
medaka LCR<>Km^r-REV	TGATGCTTAGAGGACTTCCTGGACACGGTGACAGAAAAC<br>CATCTGTCCCCTGAATCGCCCCATCATCC

Construction of the GFP reporter of the 3-kb upstream of medaka *RH2-A* and preparation of medaka RH2-LCR for co-injection

The 3-kb upstream sequence of *RH2-A* was first cloned into pBluescript II (SK-) after PCR amplification from a lambda clone derived from the HNI strain of medaka [2] using a pair of primers listed below. The forward and reverse primers had the recognition sites of EcoRI and BamHI, respectively at the 5' end. Then the amplified fragment was cut out with the restriction enzymes, and inserted into the upstream region of a GFP reporter gene of the pEGFP-1 plasmid (BD Biosciences Clontech, Tokyo, Japan). The counterpart of RH2-LCR in medaka was amplified by PCR using primer pairs listed below again from the HNI strain of medaka.

Primers for PCR amplification of the upstream sequence of medaka *RH2-A* and of the medaka RH2-LCR.

Name	Sequence (5’ > 3’)
medaka RH2-A up-3kb-FWD	TTCAGAgaattcACAAGGTTCACGCCCAGAAC
medaka RH2-A up-3kb-REV	GCCGTTggatccCTTTGCAAGTTAGGATCTGGA
medaka RH2-LCR-FWD	TACCCCGAGCTGCGAAGGAA
medaka RH2-LCR-REV	GAAATGAGCTGTTGAGTGGAAGTTATGT

Construction of reporter-expression plasmids.

The GFP expression constructs of the upstream regions of *RH2-1*, *-2*, *-3*, *-4* and *keratin 8*, attached with RH2-LCR, were constructed in the present study. pT2GFP-TKPA [3], a derivative from the plasmid clone pT2AL200R150G, which contains the Tol2 transposase recognizing sequences, L200 and R150 [5], was used as a basal vector for the construction to do Tol2 transposon-mediated transgenesis.

First, the RH2-LCR (794-bp fragment of EcoRI/EcoRI, see Figure S1B) was inserted into the EcoRI site of pT2GFP-TKPA in the same orientation as in the upstream of the RH2 gene array. Then DNA fragments consisting of the GFP encoding sequence and each of the upstream sequences of the RH2 genes were subcloned from the RH2-PAC clones in which GFP was already inserted into the positions of each of the RH2 genes. The 4.2-kb promoter of *RH2-1* with the GFP gene was obtained by BamHI/NotI digestion, and inserted in the pT2GFP-TKPA vector. The 5’ ends of the upstream regions of *RH2-2*, *RH2-3* and *RH2-4* were PCR amplified by primers

harboring SalI site on the 5' end (primers listed below), and the rest of the upstream regions together with the GFP gene was obtained from the RH2-PAC clones that have GFP replacing each of the RH2 genes through digestion by NotI, which is at the immediate downstream of the GFP coding sequence, together by another proper restriction enzyme cutting a position close to the 5' end. They were inserted in the SalI/NotI site of pT2GFP-TKPA. The *keratin 8* promoter with GFP was prepared from the LCR511:krt8 described in [1] by SalI/NotI digestion and inserted in the pT2GFP-TKPA as well.

PCR primers used for PCR-amplification of the 5' end of the upstream regions of *RH2-2*, *RH2-3* and *RH2-4*.

Name	Sequence (5' > 3')
RH2-2up3006-FWD-SalI	GCCTGTCGACATCTGCCTATTCAGTGCTCCG
RH2-3up2617-FWD-SalI	CTTTGTCGACAACTAATTGCCAACTATGCTTTC
RH2-4up7432-FWD-SalI	ATCTCGAGTCGACACTTTTTGAACTTCTTTGTGGATTA

To make the double promoter-reporter constructs with the RH2-LCR, the second reporter-promoter set was first taken out from the pT2GFP-TKPA constructs described above and inserted into the SalI/NotI site of pBluescript II (SK-). When RFP was used as a reporter for the promoters of *RH2-2* and *RH2-4*, the promoter-reporter sets were also prepared from the respective modified RH2-PAC clones. In order to put the RFP gene under the upstream sequences of *RH2-3* and keratin 8, GFP in the respective constructs above in pT2GFP-TKPA was replaced with RFP using appropriate

restriction enzymes, and the resulting constructs were inserted into the SalI/NotI site of pBluescript II (SK-). Then, into those promoter-reporter clones, the SV40-polyA sequence harboring NotI site at the 5' end that was PCR-amplified with the primers below was inserted using KpnI and SalI, which cut the sites at the 5' end of the promoter-reporter fragments in pBluescript II (SK-). As a result, (NotI)-(SV40-polyA)-promoter-reporter-(NotI) fragments were obtained for each set. Then the NotI/NotI fragment was inserted in the NotI site just upstream of HSV-TK-polyA sequence in the construct of first promoter-reporter constructs with the RH2-LCR in pT2AG-TK. As a result, constructs shown in Figures 3 and S4 were obtained.

Primers for PCR-amplification of SV40-polyA sequence.

Name	Sequence (5' > 3')
SV40-polyA-FWD	GGTACCGCGGCCGCGACTCTAGATCA
SV40-polyA-REV	CCAAGTCGACATACATTGATGAGTTTGGACAAACC

Supplemental References

1. Tsujimura T, Chinen A, Kawamura S: **Identification of a locus control region for quadruplicated green-sensitive opsin genes in zebrafish**. *Proceedings of the National Academy of Sciences of the United States of America* 2007, **104**(31):12813-12818.
2. Matsumoto Y, Fukamachi S, Mitani H, Kawamura S: **Functional characterization of visual opsin repertoire in Medaka (Oryzias latipes)**. *Gene* 2006, **371**(2):268-278.
3. Tsujimura T, Hosoya T, Kawamura S: **A single enhancer regulating the differential expression of duplicated red-sensitive opsin genes in zebrafish**. *PLoS Genet* 2010, **6**(12):e1001245.
4. Lee EC, Yu D, Martinez de Velasco J, Tessarollo L, Swing DA, Court DL, Jenkins NA, Copeland NG: **A highly efficient Escherichia coli-based chromosome engineering system adapted for recombinogenic targeting and subcloning of BAC DNA**. *Genomics* 2001, **73**(1):56-65.
5. Urasaki A, Morvan G, Kawakami K: **Functional dissection of the Tol2 transposable element identified the minimal cis-sequence and a highly repetitive sequence in the subterminal region essential for transposition**. *Genetics* 2006, **174**(2):639-649.
